# Phase behavior of single and multi-component liquid hydrocarbons in real reservoir rocks

**DOI:** 10.1038/s41598-023-31651-3

**Published:** 2023-03-18

**Authors:** Ilyas Al-Kindi, Tayfun Babadagli

**Affiliations:** Department of Civil and Environmental Eng., School of Mining and Petroleum Engineering, 7-277 Donadeo Innovation Centre for Engineering, University of Alberta, 9211 - 116th Street, Edmonton, AB T6G 1H9 Canada

**Keywords:** Energy science and technology, Energy harvesting, Fossil fuels

## Abstract

Phase-alteration phenomenon has a considerable influence on the dynamics and distribution of fluids in porous media. One of the major factors affecting the phase behaviour of fluids in reservoirs is the capillarity effect, which becomes unavoidably significant as the media becomes tighter (confinement effect) and contains more pores at nano sizes. Comprehending the nature of vaporization and condensation of hydrocarbon in such confined media is important for accurate modelling of two-phase envelopes and thereby the performance of energy production from hydrocarbon reservoirs. This paper studies the vaporization of single- and multicomponent hydrocarbons in different types of rocks (namely sandstones, limestones, tight sandstones, and shales). The vaporization temperatures were measured experimentally in each rock type and compared with boiling points measured at bulk conditions to investigate the deviation between the phase-change temperatures in capillary media and bulk values. The deviation between the measured vaporization temperatures and the bulk measurements ranged from 4.4% (1.6% in Kelvin unit) to 19.7% (5.2% in Kelvin unit) with single-component solvents and 1.4% (0.4% in Kelvin unit) to 27.6% (5.3% in Kelvin unit) with the hydrocarbon mixtures. The vaporization temperatures, obtained from the experiments, were also compared with the computed two-phase envelopes, calculated by the classical Peng-Robinson Equation of State. The deviation percentages of measured vaporization temperatures from the computed values were at least 4.4% (1.6% in Kelvin unit) with single-component solvents and 2.1% (0.7% in Kelvin unit) with the hydrocarbon mixtures.

## Introduction

One of the highly common physical phenomena in hydrocarbon reservoirs, during production or injection stages, is the fluid phase alteration due to the change of regional pressure or temperature. Throughout the production periods, the gradual pressure declines in near-wellbore areas resulting in a vaporization of lighter components over time. Injecting foreign gases in enhanced oil recovery (EOR) applications, for example, leads to a considerable phase alteration of introduced gases in the reservoir, owing to the non-isobaric conditions in the porous media. Such a phenomenon can take place frequently in high-pressure reservoirs.

One common practice in heavy-oil applications is injecting steam into the matrix to reduce oil viscosity thermally and, therefore, enhance its mobility within the capillary system. Because of the massive heat loss, steam tends to lose its heat energy and condense after injection which makes it higher in density. Generally, the occurrence of vaporization and condensation in the reservoirs is of primary importance since it impacts the dynamical behaviour of fluids, i.e. the distribution and propagation rate of fluids. Another common practice in oil reservoirs is to use liquid or gas solvents with or without thermal assistance. Change in the temperature and pressure during this type of application critically affects the hydrocarbon recovery and retrieval of expensive solvent.

Under such circumstances, it is extremely critical to accurately predict the phase change in the porous matrixes since having a desired phase can have a significant improvement on both the oil recovery and cost. A propane-injection project in tight Bakken reservoirs is a suitable example for cases which the phase of injected fluid plays a major role in achieving an ultimate oil recovery. One of the main objectives of injecting hydrocarbon solvents is reducing the heavy-oil viscosity as soon as the solvents contact the oil. Injecting the solvents in their gaseous forms is restricted by the high capillary resistance within the pores which limits the propagation of gases and contact with the oil. In Bakken reservoirs, propane was selected because of its suitable phase-change pressure that allowed it to be in liquid phase at the reservoir condition as well as its efficient contact with the oil^1^.

In simulation studies of the said applications, accurate data entry for the phase behavior of hydrocarbons is critical since it has a considerable impact on the dynamics of the processes and, therefore, oil recovery prediction. Peng and Robinson^2^ developed an equation of state (EoS) model to predict the phase behaviour of pure-component and multicomponent fluids, including other physical properties, such as densities and volumetric behaviours:

1 $$P=\frac{RT}{{V}_{m}-b}-\frac{a \left(T\right)}{{V}_{m}\left({V}_{m}+b\right)+b\left({V}_{m}-b\right)},$$

where $R$ is universal gas constant (8.31 J/mol.K), $T$ is fluid temperature (a range between 293.15 and 473.15 K), ${V}_{m}$ is molar volume of the fluid, based on system’s pressure and temperature. Table 1 shows the molar volumes of used pure components at 293.15 K (20 °C). $a (T)$ and $b$ are attraction parameter and van der Waals co-volume which their quantities are subjected to pressure and temperature.

**Table 1:** Molar volumes of pure hydrocarbons used in the experiments.

Hydrocarbon component	Molar volume (mL/mol)
n-Pentane (C_5_)	115
n-Heptane (C_7_)	147.5
n-Octane (C_8_)	163.5

Earlier, Redlich and Kwong^3^ proposed an equation which linked the variation of pressure with volume and temperature. The equation was, then, modified by Soave^4^ to enhance the accuracy of the previous EOS model^3^. The original forms of the Peng Robinson (PR) and Redlich Kwong (RK) cubic EoS do not take the capillary pressure and adsorption effect into account. However, it has recently been shown that neglecting these factors may cause the PR-EoS and RK-EoS to yield inaccurate estimation of phase behavior in confined porous media, especially when consisting of a larger percentage of nanopores^5–7^.

Several studies^8–12^ were conducted to apply the notion of capillary (or confinement) effect in the original cubic EoS. Travalloni et al.^13^ developed an extended version of PR-EoS which considered the capillarity and pore-molecule effects on the phase behaviour of confined fluids. Nojabaei et al.^14^ included the capillary pressure in vapour-liquid equilibrium (VLE) calculations and then, adopted to PR-EoS to compute the pressure–temperature phase envelopes for a number of binary mixtures. Considering the capillarity effect in the VLE equations led to a reduction in the bubble point pressure and an increase/decrease in the dew point pressure. As the medium becomes tighter, constrained fluids start to get highly affected by the pore wall (molecule-pore interaction), owing to the limited number of molecules^15^ which results in a heterogeneous fluid distribution^16^. Cui et al.^15^ introduced a modified version of PR-EoS by adjusting the molar volume term based on the fluid reduced mole number, of which is resulted by the adsorption phenomenon.

Comprehending the phase-change behaviour of fluids in porous media is an important aspect to accurately predict fluids’ dynamics, phase distribution, and oil/gas recoveries. The issue becomes more critical in tight reservoirs (shale, tight sandstone, etc.) since such rocks are mainly governed by nanopores^17,18^. Shifted phase-alteration temperatures and pressures of fluids become observable once the medium sizes go below 1000 nm (nm) as stated by the classical Kelvin equation^19^ or 100 nm as reported by Cui et al.^15^.

In a previous study^18^, pore size distribution analysis was performed to identify the deviation of pore sizes in shale, tight sandstone, Indiana limestone, and Berea sandstone. The analysis showed that around 4% of the channels in the permeable rocks (sandstone and limestone) were smaller than 100 nm. As a result, shifted vaporization pressures of propane, heptane, and octane were noticed in the permeable rocks, including the tight rocks (shale and tight sandstone) at which a high percentage of their pore diameters are in nanoscale (< 100 nm). This paper is a continuation of our previous works^5–7,18,20^, and it investigates the vaporization temperature of pure-component hydrocarbon solvents, binary mixtures, and ternary mixtures in different reservoir rocks and under various pressures. Experimental data and evidences are provided as to the effect of confined environment (nano-pores and high capillary pressures) on the phase behavior (vaporization) of solvents. The experimental outcomes are then compared with computed vaporization temperatures obtained by the classical PR-EoS.

## Problem statement and solution methodology

**Figure 1:**
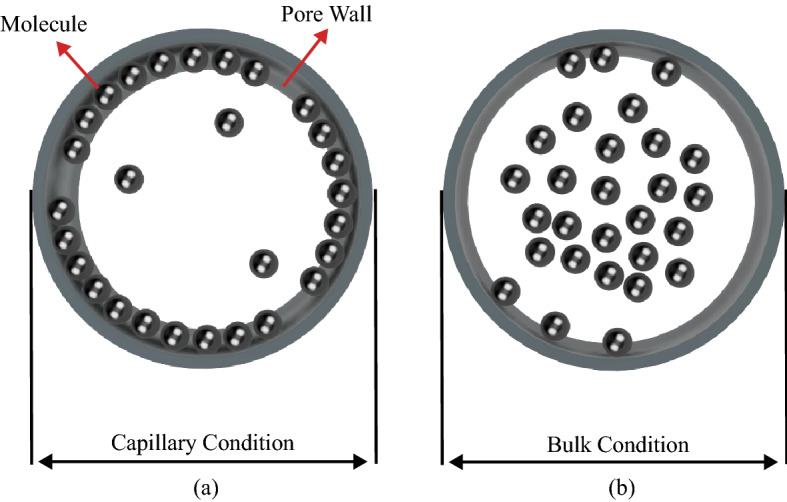
(**a**) Distribution of fluid molecules in a tight pore. Due to the confinement effect, most of the molecules are adsorbed by the inner pore surface; (**b**) distribution of fluid molecules in a bulk medium with no capillary effect. The minority of the molecules are adsorbed by the solid surface since the cohesion forces (intermolecular forces) are the dominant.

Confined fluids inherit distinctive physical properties different from those existing in bulk conditions^21^; consequently, the confinement effects tend to alter the phase-change behaviour of constrained fluids^22–24^. In bulk conditions, the behaviour of phase transition is mainly governed by molecule–molecule interactions (intermolecular bonds), and the majority of the fluid molecules are not impacted by the adhesion forces, due to the solid-molecule interaction. The number of molecules in the space volume reduces as the medium size gets smaller. As a consequence, the influence of adhesion forces (pore-molecule interaction) on a large portion of molecules begins to take place and introduces molecule adsorption on the pore wall. This phenomenon contributes in altering the phase-change temperature/pressure of constrained fluids^13^. Moreover, the molecule adsorption in nanopores results in a heterogeneity of fluid density distribution that could be the cause of shifted phase behaviours^25^. Figure 1 illustrates a schematic representation of molecules’ interactions with the pore wall in the bulk and capillary conditions.

On the other hand, the phase change of the fluids in the reservoir has a significant control on fluid dynamics in porous media and, therefore, influences the distribution of phases in the reservoir, propagation of reservoir gases and injected solvents, and overall hydrocarbon recoveries. Injecting high-temperature fluids (i.e. steam, hot water), injecting solvents as an additive to steam^26,27^, or during reservoir pressure depletion, vaporization or condensation of contacted reservoir and injected fluids continuously occurs throughout the production or injection stages. Tight reservoir rocks are mainly characterized with extended tightness, introducing a significant capillary effect on the phase behaviour. Hence, understanding thoroughly the impact of capillarity on the nature of phase change is essential to achieve accurate modeling of fluid properties and conduct precise VLE calculations for confined fluids in tight reservoirs. Standard cubic EoS’s do not consider the effect of confinement on the phase change which leads them not to be suitable candidates for achieving precise VLE calculations for confined fluids in tight media.

Besides the improved accuracy of phase dynamic predications, modelling the phase behaviour precisely in reservoir rocks is critical in high-temperature EOR applications (i.e. steam injection) to estimate the right temperatures for attaining optimum hydrocarbon recoveries. For instance, knowing the right temperature of the injected steam/hot water in Solvent-Over-Steam Injection in a Fractured Reservoir (SOS-FR) is important to reach the optimal recovery of injected solvents^27^. The correct estimation of temperature is beneficial in reducing the operational cost by eliminating the need for excessive heat energy. Pore size distribution analysis (PSDA) was conducted previously in our prior works^6–18^ to experimentally measure the distribution of pore sizes in several reservoir rocks. These studies showed that nearly 4.5% of the pore volume in permeable rocks (Berea sandstone and Indiana limestone) are of nano-size, despite their relatively high permeabilities. The existence of nanopores could result in shifted vapour pressures or boiling temperatures in the permeable rocks, although the volume percentage of confined pores (< 1000 nm) is considerably minor.

The tested single-component solvents (heptane and octane) represented the injected hydrocarbon solvents in cold solvent injection or thermal EOR application at which solvents are used as additives to steam. The binary mixture (pentane-heptane) represented a non-complex light oil; meanwhile, the ternary mixture acted as a heavier oil with a slightly more complexity in terms of components, comparing with the binary mixture. The shifted boiling temperatures were measured under three main pressures: (a) atmospheric pressure (1 atm, 14.7 psi); (b) 64.7 psi; (c) 114.7 psi. A special high-pressure-high-temperature (HPHT) windowed cell was used to perform the analysis and kept in a constant-temperature oven to control the temperature of the windowed cell and, specifically, the rocks in the cell. At vaporization temperatures, the generated vapour bubbles were detected using a video camera, featured with a magnification system which provided a clear visualization of the micro vapour bubbles on the outer rock sample. The shifted boiling temperatures were compared with computed vaporization temperatures by PR-EoS, and deviation percentages were calculated between the measured values from the experiments and computed values from the cubic EoS.

## Experimental design and methodology

**Figure 2:**
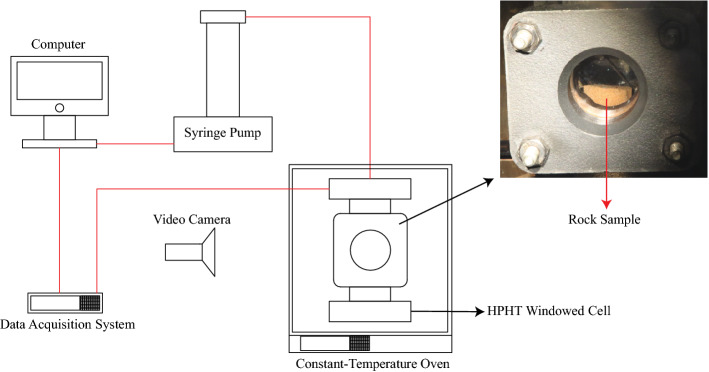
A schematic of the experimental system utilized for solvent vaporization temperature measurement. The rock sample was immersed in glycerol which acted as a heating liquid. The thermocouple was also immersed in glycerol to measure its temperature continuously.

The HPHT cell shown in Fig. 2 was pressurized at constant pressures by an inert gas. Nitrogen was selected as the pressurizing gas since it is chemically inactive with the fluids inside the cell at the condition of our experiments, and it is generally one of the major pre-existing gases in oil and gas reservoirs. The applicable pressure range was 14.7–114.7 psi based on the maximum design temperature of the windowed cell, which was 260 °C (533.15 K). Going beyond 114.7 psi would require us to exceed the maximum design temperature in order to approach the bulk phase-change temperatures of the used solvents.

The pressure of the system was controlled by a syringe pump, featured with a standard pressure accuracy of 99.5%. The overall cell temperature was raised gradually from the room temperature (21 °C) to the bulk boiling temperature of the tested solvent, either a pure-component liquid or mixture, by using the constant-temperature oven. In all the experiments, temperatures were increased with a heating rate of 0.05 °C/sec. Before initiating the trials, selected rocks were vacuumed at 12 psi (below atmospheric pressure) to thoroughly remove the trapped air. Then, they were saturated with the hydrocarbon solvents for at least 24 h to insure a complete saturation in each rock sample. One of the critical points in the investigation is to guarantee a uniform distribution of heat around the rock. To do so, the rock samples were immersed in a glycerol bath which acted as a heating liquid. Glycerol, as a water-soluble liquid, is not soluble in non-polar hydrocarbon solvents; hence, such a chemical property would prevent the tested solvents to mix with glycerol, forming a solution. Other reasons why glycerol was chosen to be the medium for proper (uniform) heating of the rock is that it was very difficult for glycerol to get imbibed into the rock pores due to its high viscosity and as the rock samples were carefully vacuumed to remove completely the trapped air and fully saturated with hydrocarbons. On the other hand, even if the glycerol has an access to the rock pores, its boiling temperature (~ 290 °C) is higher than the designed maximum temperature of the experiments which was 180 °C. Hence, the glycerol did not encounter a phase alteration or interaction with the rock even at the maximum temperature conditions of our experiments.

Figure 2 illustrates schematically the experimental setup used to study the phase behaviour of hydrocarbon solvents in reservoir rocks. By using a data acquisition system, the pressure and temperature of HPHT cell, including the rocks, were recorded every second with the assistance of an immersion thermocouple and a pressure transducer. The low heating rate (0.05 °C/sec) was chosen to prevent any temperature difference between the heating liquid (glycerol) and rock sample during the heating process. Thus, the temperature of glycerol was recorded continuously along with the pressure. At vaporization stages, the formed vapour phase was detected by a video camera to capture the formation of gas bubbles on the rock surface. System calibration was done to minimize any possible measurement error. Vaporization at bulk conditions was used as a benchmark to calibrate the setup and inspect potential inaccuracies that could be caused by the sensors or heat heterogeneity in the system. The normal boiling points of tested liquids were measured using the experimental system (Fig. 2) and compared with standardized phase-change temperatures that were measured under identical conditions in terms of volume and pressure.

### Pore size distribution analysis (PSDA)

In our previous studies^6–18^, PSDA was performed to measure deviation of pore sizes in Berea sandstone, Indiana limestone, tight sandstone, and shale. The study showed several measured parameters including occupation percentage of pores tighter than 1000 nm in each rock. The analysis of pore size distribution was done by quantifying the nitrogen desorption and adsorption on the rock surface. Table 2 presents volume percentages of nanopores (< 1000 nm), permeability, density in sandstone, limestone, tight sandstone, and shale.

**Table 2:** Permeability, density, and pore volume percentages in various tested reservoir rocks (Al-Kindi and Babadagli^6^).

Rock type	Permeability (md)	Density (kg ${m}^{-3}$)	Volume percentage of pores tighter than 1000 nm (%)
Berea sandstone	274	2129	4.4
Indiana limestone	30	2246	4.6
Tight sandstone	0.1	2400	38.2
Shale	< 0.01	2200	94.3

The high-permeability rocks (sandstone and limestone) mainly consist of macropores and nearly 95.5% of the pores are larger than 1000 nm, according to the PSDA. Based on the literature, the phase behaviour of fluids inside the majority of the pores in the permeable rocks could be possibly predicted by the classical PR-EoS since the capillary effect would not be sufficient to cause any alteration to boiling temperatures. Nonetheless, fluids in confined pores, which act as minority in sandstone and limestone, would behave differently in terms of phase alteration due to high capillary pressures and adsorption effects. Also, the shifted boiling temperatures would not be modelled accurately by conventional cubic EoS, such as PR-EoS and RK-EoS. In tight matrixes (shale and tight sandstone), modelling the phase-change behaviour of hydrocarbons is extremely critical since confined pores (< 1000 nm) occupy the tight rocks at considerable percentages.

### Materials

The investigation was initiated by studying the vaporization behaviour of single-component solvents (heptane and octane). Then, the study focused on more complicated mixtures with binary and ternary hydrocarbon components. Table 3 shows the mole fraction and purity of the used alkanes.

**Table 3:** Purity and mole fraction of the alkanes used in the experiments.

Hydrocarbon Component	Purity of each Component	Mole fraction
Heptane	Heptane: 99.7%	Heptane: 100%
Octane	Octane: 99.8%	Octane: 100%
Pentane-heptane	Pentane: 99.7%	Pentane: 50%
	Heptane: 99.7%	Heptane: 50%
Pentane-heptane-octane	Pentane: 99.7%	Pentane: $\frac{100}{3}$%
	Heptane: 99.7%	Heptane: $\frac{100}{3}$%
	Octane: 99.8%	Octane: $\frac{100}{3}$%

### Quantitative analysis

The PR-EoS was used to model two-phase envelopes for the single-component and multicomponent hydrocarbon solvents. The two-phase envelopes were generated using the Computer Modelling Group (CMG) software. The selected ranges of temperature and pressure for the phase envelope calculations were 0 °C (273.15 K)–250 °C (523.15 K) and 0 psi (0 bar)–200 psi (13.7 bar). The mean phase-change temperatures in the rocks were then compared with those computed by the cubic EoS. For mixtures, the measured temperatures were compared with the bubble point temperatures calculated by the PR-EoS. For each trial, the deviation percentage ${(\Delta T}_{v}\%)$ between the shifted temperature values and calculated values from the PR-EoS was obtained, and it was expressed as following:

2 $$\Delta {T}_{v}\%=\left|\frac{{T}_{v}- {T}_{exp}}{{T}_{v}}\right|\times 100,$$

in which ${T}_{v}$ is the computed bubble point temperature by the PR-EoS at the given pressure, and ${T}_{exp}$ is the measured vaporization temperature in the rock. Table 4 (see Supplementary Material section) presents the measured solvent boiling temperature in each rock, at various surrounding pressures, and the deviation percentage ($\Delta {T}_{v}\%$) between the temperatures obtained from the experiments and those modelled by the PR-EoS.

## Experimental results

Owing to the heterogeneous nature of reservoir rocks, the motion of vapour phase inside the rock porous medium would slightly change in every trial, meaning that the temperature at which the generated bubble would appear on the rock surface could marginally vary. Therefore, experiments were repeated more than once to obtain representative values by averaging the measured boiling temperatures. The mean values of phase-change temperatures at various pressures in bulk conditions were considered in the analysis as shown in Figs. 4, 5, 6, and 7. The values for bulk conditions were obtained from literature or other sources (catalogs etc.) and highly comparable values were observed between the PR-EoS and bulk measurement of vaporization temperatures as seen in these plots. The measured phase-change temperatures of solvents in the reservoir rocks were different from the bulk measurement and calculated values from PR-EoS.

**Figure 3:**
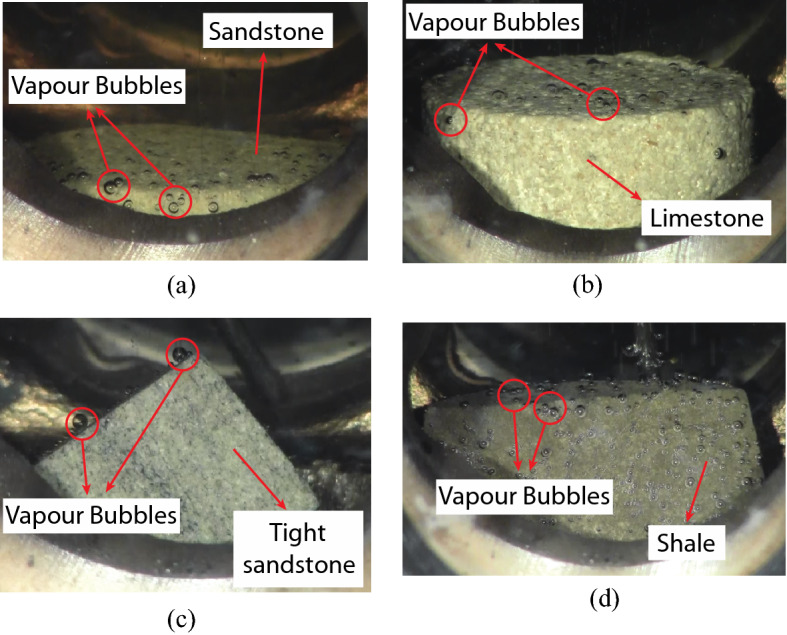
(**a**) Vaporization of pure heptane in sandstone at 137 °$\mathrm{C }$and 64.7 psi; (**b**) vaporization of pure octane in limestone at 163 °$\mathrm{C}$ and 64.7 psi; (**c**) vaporization of pentane-heptane mixture in tight sandstone at 134 °$\mathrm{C}$ and 114.7 psi; (**d**) vaporization of pentane-heptane-octane mixture in shale at 127 °$\mathrm{C}$ and 64.7 psi.

Figure 3 shows the generation of vapour phase of pure-component solvents and hydrocarbon mixtures in different reservoir rock types and pressures. Table 5 (see Supplementary Material section) presents the measured solvent boiling temperature in each rock, at each selected pressure, and the deviation percentage $(\Delta {T}_{B}\%$) between the mean temperatures obtained from the experiments and those measured in bulk conditions, given as follows:

3 $$\Delta {T}_{B}\%=\left|\frac{{T}_{B}- {T}_{exp}}{{T}_{B}}\right|\times 100,$$

where ${T}_{B}$ is the boiling point of solvent at bulk condition, and ${T}_{exp}$ is the experimental vaporization temperature in the rock. With pure hydrocarbon solvents (heptane and octane), the deviation percentages were observed to be varying from 4.4 (1.6% in Kelvin unit) to 19.7% (5.2% in Kelvin unit), due to the existence of confined pores in the reservoir rocks. Meanwhile, with hydrocarbon mixtures, the deviation percentages were observed to be varying from 1.4 (0.4% in Kelvin unit) to 27.6% (5.3% in Kelvin unit).

**Figure 4:**
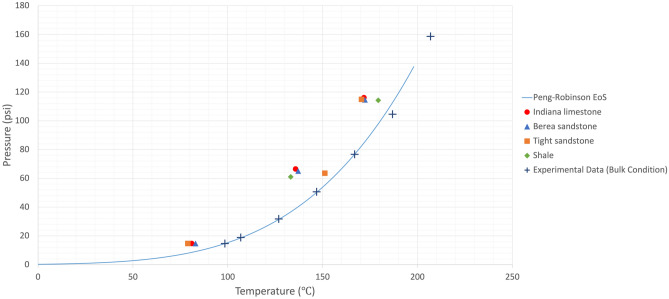
Mean vaporization temperatures of heptane in various rocks and computed phase-change temperatures obtained from the PR-EoS at different pressures. The experimental data were obtained from the phase-change measurement at bulk conditions.

**Figure 5:**
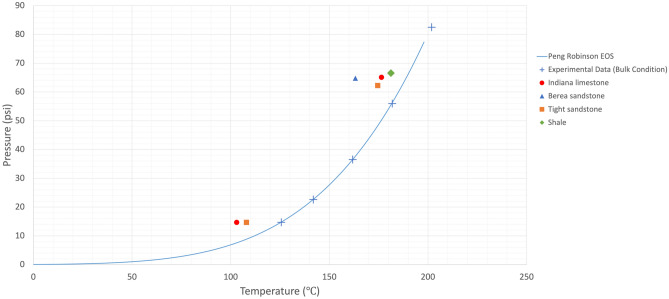
Mean vaporization temperatures of octane in various rocks and computed phase-change temperatures obtained from the PR-EoS at different pressures. The experimental data were obtained from the phase-change measurement at bulk conditions.

**Figure 6:**
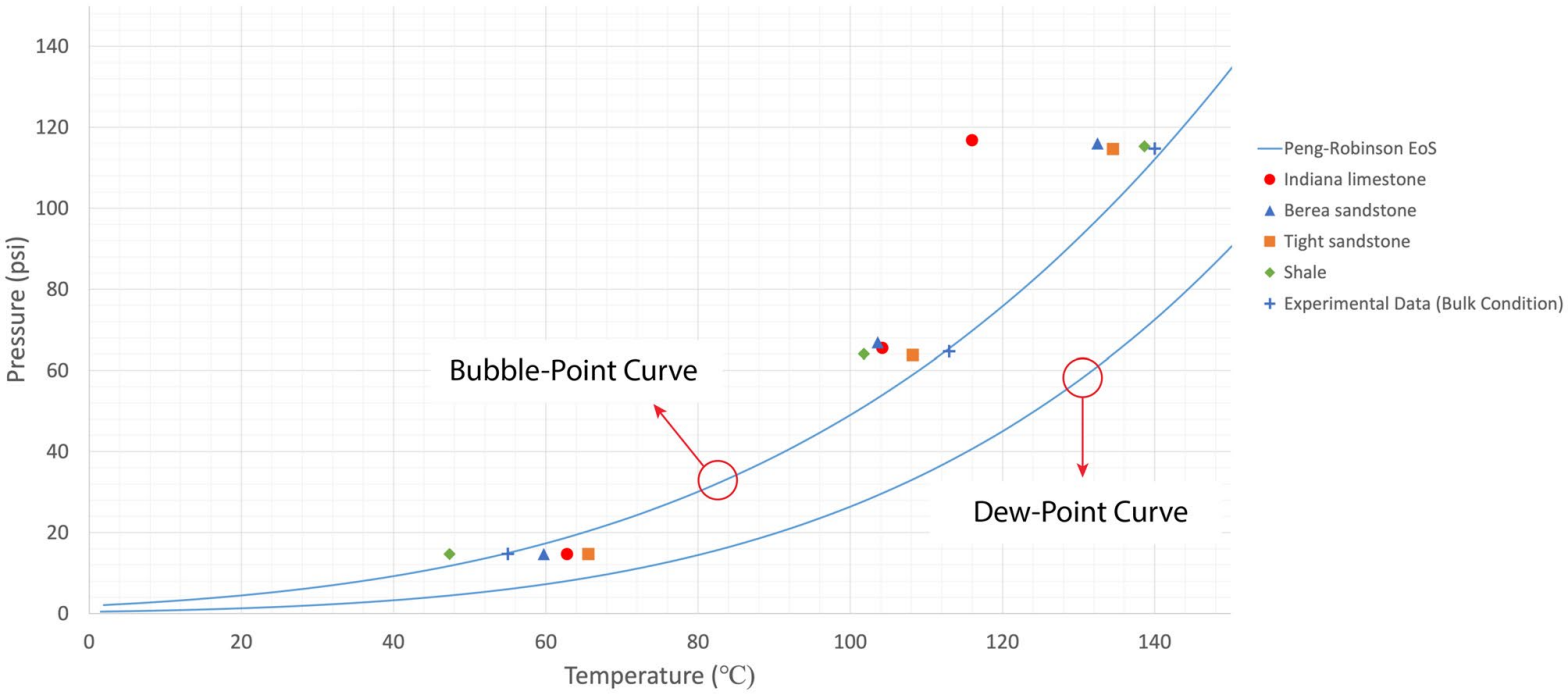
Mean vaporization temperatures of pentane-heptane mixture in various rocks and computed bubble-point/dew-point temperatures obtained from the PR-EoS at different pressures. The experimental data were obtained from the phase-change measurement at bulk conditions.

**Figure 7:**
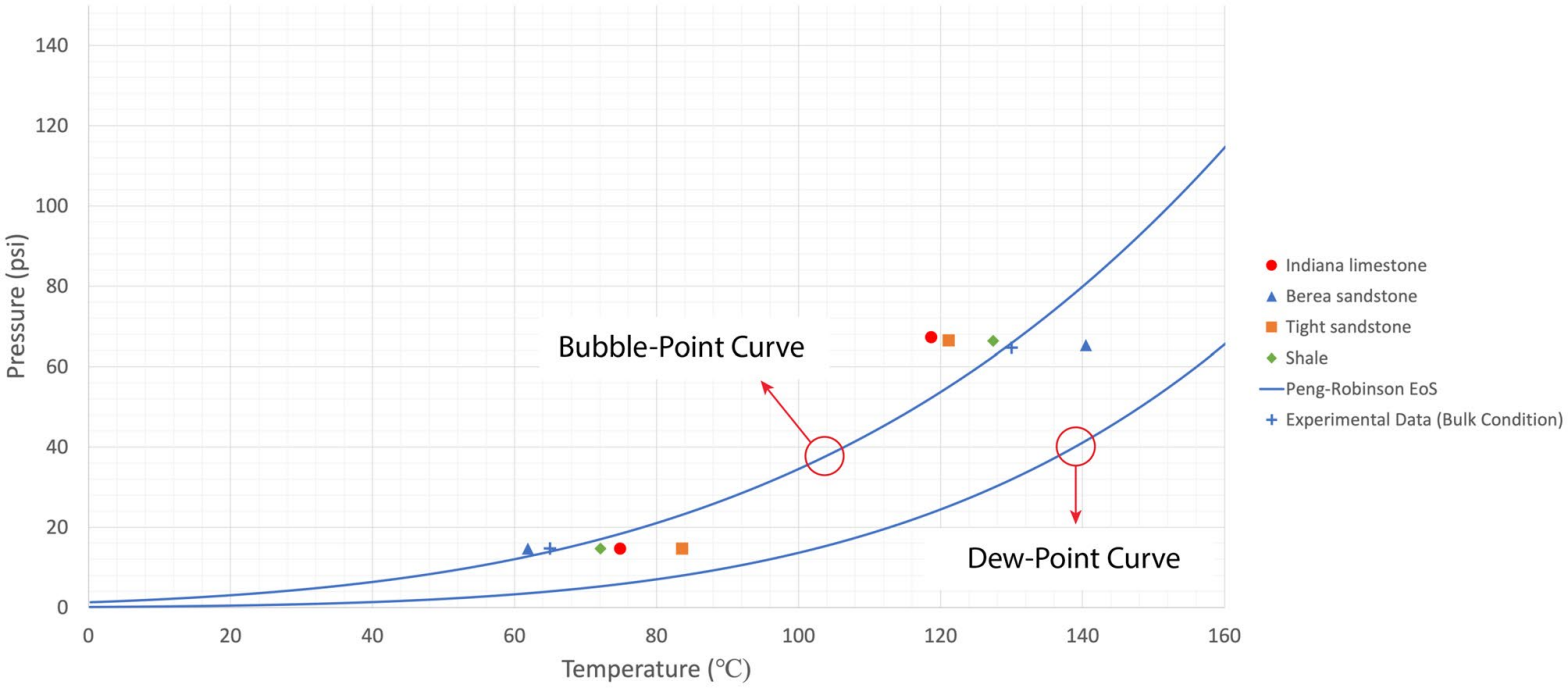
Mean vaporization temperatures of pentane-heptane-octane mixture in various rocks and computed bubble-point/dew-point temperatures obtained from the PR-EoS at different pressures. The experimental data were obtained from the phase-change measurement at bulk conditions.

Figures 4 and 5 show the measured vaporization temperatures of heptane and octane, respectively, as pure-component solvents in sandstone, limestone, tight sandstone, and shale. Figures 6 and 7 display the measured vaporization temperatures of pentane-heptane mixture and pentane-heptane-octane mixture in sandstone, limestone, tight sandstone, and shale, respectively. Furthermore, they present the computed two-phase envelopes of the tested solvents using the classical PR-EoS, including the bulk boiling temperatures of each solvent which were measured experimentally at bulk conditions with no capillary effects. Owing to the capillary effect in the rocks, the deviation percentages between the measured and calculated phase-change temperatures were ranging from 4.4 (1.6% in Kelvin unit) to 19.3% (5.1% in Kelvin unit) with pure solvents (heptane and octane). Whereas, with multicomponent solvents, the deviation percentages were ranging from 2.1 (0.7% in Kelvin unit) to 25.7% (5% in Kelvin unit).

## Discussion

**Figure 8:**
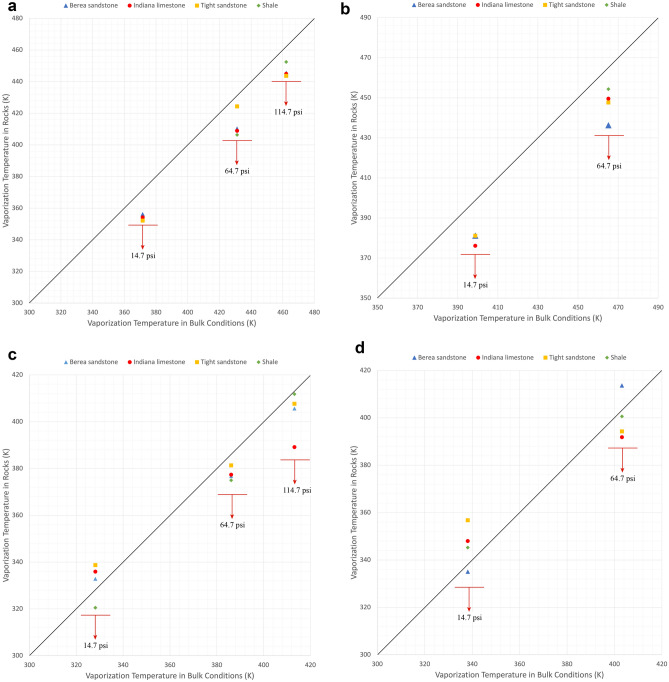
(**a**) Deviation of measured vaporization temperatures of heptane from bulk measurements. (**b**) Deviation of measured vaporization temperatures of octane from bulk measurements. (**c**) Deviation of measured vaporization temperatures of pentane-heptane mixture from bulk measurements. (**d**) Deviation of measured vaporization temperatures of pentane-heptane-octane mixture from bulk measurements.

Figure 8a–d illustrate the deviation of experimentally measured vaporization temperatures in the rocks from the bulk measurements of heptane, octane, binary mixture (pentane-heptane), and ternary mixture (pentane-heptane-octane). The continuous lines represent a zero deviation between the bulk values and temperature measurements in the rocks. With pure-component solvents (Fig. 8a,b), the shift of measured vaporization temperatures from the bulk measurements was observed at all selected pressures and rock types. Systematically, the temperature required for a given pressure to start the boiling is lower than that of bulk conditions with no exception. Meanwhile, with binary and ternary mixtures, relatively minor deviations were detected at the atmospheric pressure (Fig. 8c,d). As the pressure increased, the difference between the measured and bulk values increased. The temperatures required for boiling was observed to be lower than the bulk condition at higher pressures (Fig. 8c,d) as similar to the single component cases (Fig. 8a,b).

The capillary characteristics of the rocks such as permeability, pore texture, wettability, and clay content may also play a role in the boiling process. All these parameters affect the phase distribution and entrapment of generated gas, which eventually results in different phase behavior in multi-component systems. In situations where a single-component solvent exists, at the phase-change stage, a large percentage of the vapour phase does not get interrupted or trapped by the liquid phase from the same fluid^20^. This phenomenon explains the cause of noticeable temperature deviations of pure solvents from their normal boiling points (Fig. 8a,b). On the other hand, with multicomponent solvents, the liquid phase of heavier components with higher boiling temperatures restricts the movement of the vapour phase of lighter components as observed in our previous study^20^ in silicate glass microfluidic chips. The delay of vapour bubbles to appear on the rock surface results in reduction of the temperature deviations specifically at the atmospheric pressure. Hence, the impact of rock -interfacial- properties is more pronounced in case of multi-component systems, and this requires further research.

Most of the earlier studies on the capillary effect on the phase behavior were done for isothermal conditions and changing pressure. These studies are limited to theoretical and computation developments with limited experimental verifications. They concluded that lower pressures are needed compared to the bulk conditions for vaporization, which is in line with the Kelvin equation^6,7^. In these studies, hydrocarbon gases (typically propane) were used. In the present study, however, we tested liquid hydrocarbons and non-isothermal conditions. This is a common case in oil reservoirs in which solvents were used with steam to enhance oil recovery. The minimum temperature requirement for this type of applications is a critical problem^27^ but the theoretical models and, more critically, experimental data are rare for this type of applications. The tendency was to use liquid hydrocarbons (pentane-decane range) rather than gaseous ones (propane-butane) for more efficient mixing and less asphaltene precipitation.

Experimental evidences for this type of hydrocarbons are rare. Alfi et al.^28^ tested hexane, heptane, and octane on nanofluidic chips at variable temperatures. Cho et al.^29^ used mesoporous silica materials for a decane-methane mixture. Both studies reported very slight increase in bubble point temperatures, which is in line with the Kelvin equation. The lack of experimental data is a critical problem on this complex phenomenon as concluded by Barsotti et al.^22^ after their extensive review on the capillary effect on the phase behavior. They indicated that there are still many unknowns on the capillary condensation/vaporization and confinement induced phase transition. This requires more experimental evidences.

With the efforts on rock experiments, we provided substantial experimental evidences. The data is contradictory to the theories (Kelvin equation) at certain conditions especially at higher temperatures and pressures (Figs. 4, 5, 6 and 7). This could be attributed to the medium characteristics. Rocks are more complex than micro/nano chips as they represent a wide range of capillary sizes with complex network characteristics, wettability characteristics and mineralogical factors (clay contents etc.). This requires further clarifications and it is hoped that the experimental data provided in this work will be useful in leading to further experimental and computational studies.

## Conclusions and remarks

Understanding the phase behaviour in reservoir rocks is essential to achieve precise predictions of fluids’ dynamics and distributions in porous media. The impact of confinement effect on vaporization and condensation behaviors becomes more pronounced in tight reservoirs, such as shale and tight sandstone matrixes. The existence of nanopores in permeable rocks (Berea sandstone and Indiana limestone) could also result in shifted phase-change temperatures, as observed in Table 4 and Figs. 4, 5, 6, and 7. The deviation percentages of measured vaporization temperatures in all the rock samples from the bulk measurements ranged from 4.4% (1.6% in Kelvin unit) to 19.7% (5.2% in Kelvin unit) with single-component solvents and 1.4% (0.4% in Kelvin unit) to 27.6% (5.3% in Kelvin unit) with the hydrocarbon mixtures. The shifted phase-change temperatures were also compared with the modelled two-phase envelopes by the original version of PR-EoS, as observed in Table 5 and Figs. 4, 5, 6 and 7. The deviation percentages of measured vaporization temperatures from the computed values were at least 4.4% (1.6% in Kelvin unit) with single-component solvents and 2.1% (0.7% in Kelvin unit) with the hydrocarbon mixtures.

The aim of this paper was to show how the phase behavior of hydrocarbon mixtures in real reservoir rocks (capillary -confined- media) deviates from the bulk conditions. This was achieved through an experimental study. Our future study will focus on the modification of the original version PR-EoS to make it more applicable in modelling the phase-change behaviour in confined porous media. The experimental data provided in this work would be useful in the validation of such analytical/computational models.

## Supplementary Information

Supplementary Tables.

## Data Availability

The datasets used and/or analysed during the current study available from the corresponding author on reasonable request.

## List of symbols

PR-EoS: Peng Robinson equation-of-state
EOR: Enhanced oil recovery
PR: Peng Robinson
RK: Redlich Kwong
VLE: Vapour-liquid equilibrium
$R$: Universal gas constant
$$T$$: Temperature
$$a (T)$$: Attraction parameter
$${V}_{m}$$: Molar volume
$b$: Van der Waals co-volume
$$Z$$: Compressibility factor
$$A$$, $$B$$: Constants
$$P$$: Pressure
PSDA: Pore size distribution analysis
HPHT: High-pressure-high-temperature
K: Kelvin
$$^\circ{\rm C}$$: Degree celsius
Sec: Second
Psi: Pound per square inch
$\Delta {T}_{B}\%$ and $\Delta {T}_{v}\%$: Deviation percentages
${T}_{B}$: Boiling point of solvent at bulk condition
${T}_{exp}$: Experimental vaporization temperature in the rock
${T}_{v}$: Computed bubble point temperature by the PR-EoS
$$\%$$: Percentage
C: Carbon atom
H: Hydrogen atom
C_7_H_16_: Heptane
C8H18: Octane
SOS-FR: Solvent-over-steam injection in fractured reservoirs
