# Supplementary material for: Phase behavior of single and multi-component liquid hydrocarbons in real reservoir rocks
Source: Sci Rep. 2023 Mar 18;13:4507. doi: 10.1038/s41598-023-31651-3 (PMC10024710; doi:10.1038/s41598-023-31651-3)
Supplement: Supplementary file 1 — Supplementary Tables. [file 41598_2023_31651_MOESM1_ESM.docx]

**Supplementary Material**

**Table 4–Shifted vaporization temperatures of several hydrocarbon liquids in different reservoir rocks and at various surrounding pressures (14.7, 64.7, and 114.7 psi). The bulk vaporization temperatures were obtained from a phase-change experimental analysis at bulk conditions.**

| Heptane (C_7_H_16_) – 14.7 psi | | | | | |
| --- | --- | --- | --- | --- | --- |
| Rock Type | Experimental Vaporization Temperature ($℃$) – Trial 1 | Experimental Vaporization Temperature ($℃$) – Trial 2 | Mean Experimental Vaporization Temperature ($℃$) | Bulk Vaporization Temperature ($℃$) | Deviation Percentage (%) |
| Berea sandstone | 84 | 82 | 83 | 98.4 | 15.6 |
| Indiana Limestone | 79 | 83 | 81 | 98.4 | 17.6 |
| Tight sandstone | 81 | 77 | 79 | 98.4 | 19.7 |
| Heptane (C_7_H_16_) – 64.7 psi | | | | | |
| Berea sandstone | 138 | 136 | 137 | 158 | 13.2 |
| Indiana Limestone | 134 | 136 | 135 | 158 | 14.5 |
| Tight sandstone | 148 | 154 | 151 | 158 | 4.4 |
| Shale | 132 | 134 | 133 | 158 | 15.8 |
| Heptane (C_7_H_16_) – 114.7 psi | | | | | |
| Berea sandstone | 171 | 173 | 172 | 189 | 8.9 |
| Indiana Limestone | 173 | 169 | 171 | 189 | 9.5 |
| Tight sandstone | 169 | 171 | 170 | 189 | 10 |
| Shale | 181 | 177 | 179 | 189 | 5.2 |
| Octane (C8H18) – 14.7 psi | | | | | |
| Berea sandstone | 105 | 101 | 103 | 125.6 | 17.9 |
| Indiana Limestone | 107 | 109 | 108 | 125.6 | 14 |
| Tight sandstone | 106 | 110 | 108 | 125.6 | 14 |
| Octane (C8H18) – 64.7 psi | | | | | |
| Berea sandstone | 178 | 174 | 176 | 192 | 8.3 |
| Indiana Limestone | 162 | 164 | 163 | 192 | 15.1 |
| Tight sandstone | 173 | 175 | 174 | 192 | 9.3 |
| Shale | 183 | 179 | 181 | 192 | 5.7 |
| Pentane-heptane mixture – 14.7 psi | | | | | |
| Berea sandstone | 61 | 57 | 59 | 55 | 7.2 |
| Indiana Limestone | 61 | 63 | 62 | 55 | 12.7 |
| Tight sandstone | 63 | 67 | 65 | 55 | 18.1 |
| Shale | 48 | 46 | 47 | 55 | 14.5 |
| Pentane-heptane mixture – 64.7 psi | | | | | |
| Berea sandstone | 105 | 101 | 103 | 113 | 8.8 |
| Indiana Limestone | 103 | 105 | 104 | 113 | 7.9 |
| Tight sandstone | 105 | 111 | 108 | 113 | 4.4 |
| Shale | 103 | 99 | 101 | 113 | 10.6 |
| Pentane-heptane mixture – 114.7 psi | | | | | |
| Berea sandstone | 133 | 131 | 132 | 140 | 5.7 |
| Indiana Limestone | 115 | 117 | 116 | 140 | 17.1 |
| Tight sandstone | 133 | 135 | 134 | 140 | 4.2 |
| Shale | 137 | 139 | 138 | 140 | 1.4 |
| Pentane-heptane-octane mixture – 14.7 psi | | | | | |
| Berea sandstone | 62 | 60 | 61 | 65 | 6.1 |
| Indiana Limestone | 75 | 73 | 74 | 65 | 13.8 |
| Tight sandstone | 81 | 85 | 83 | 65 | 27.6 |
| Shale | 70 | 74 | 72 | 65 | 10.7 |
| Pentane-heptane-octane mixture – 64.7 psi | | | | | |
| Berea sandstone | 141 | 139 | 140 | 132 | 6 |
| Indiana Limestone | 119 | 117 | 118 | 132 | 10.6 |
| Tight sandstone | 120 | 122 | 121 | 132 | 8.3 |
| Shale | 125 | 129 | 127 | 132 | 3.7 |

**Table 5–Shifted vaporization temperatures of several hydrocarbon liquids in different reservoir rocks and at various surrounding pressures (14.7, 64.7, and 114.7 psi). The calculated vaporization/bubble point temperatures were obtained from the PR-EoS.**

| Heptane (C_7_H_16_) – 14.7 psi | | | |
| --- | --- | --- | --- |
| Rock Type | Experimental Vaporization Temperature ($℃$) | Computed Vaporization\Bubble Point Temperature ($℃$) | Deviation Percentage (%) |
| Berea sandstone | 83 | 98 | 15.3 |
| Indiana Limestone | 81 | 98 | 17.3 |
| Tight sandstone | 79 | 98 | 19.3 |
| Heptane (C_7_H_16_) – 64.7 psi | | | |
| Berea sandstone | 137 | 158 | 13.2 |
| Indiana Limestone | 135 | 158 | 14.5 |
| Tight sandstone | 151 | 158 | 4.4 |
| Shale | 133 | 158 | 15.8 |
| Heptane (C_7_H_16_) – 114.7 psi | | | |
| Berea sandstone | 172 | 188 | 8.5 |
| Indiana Limestone | 171 | 188 | 9 |
| Tight sandstone | 170 | 188 | 9.5 |
| Shale | 179 | 188 | 4.7 |
| Octane (C8H18) – 14.7 psi | | | |
| Berea sandstone | 103 | 126 | 18.2 |
| Indiana Limestone | 108 | 126 | 14.2 |
| Tight sandstone | 108 | 126 | 14.2 |
| Octane (C8H18) – 64.7 psi | | | |
| Berea sandstone | 176 | 188 | 6.3 |
| Indiana Limestone | 163 | 188 | 13.2 |
| Tight sandstone | 174 | 188 | 7.4 |
| Shale | 181 | 188 | 3.7 |
| Pentane-heptane mixture – 14.7 psi | | | |
| Berea sandstone | 59 | 56 | 5.3 |
| Indiana Limestone | 62 | 56 | 10.7 |
| Tight sandstone | 65 | 56 | 16 |
| Shale | 47 | 56 | 16 |
| Pentane-heptane mixture – 64.7 psi | | | |
| Berea sandstone | 103 | 114 | 9.6 |
| Indiana Limestone | 104 | 114 | 8.7 |
| Tight sandstone | 108 | 114 | 5.2 |
| Shale | 101 | 114 | 11.4 |
| Pentane-heptane mixture – 114.7 psi | | | |
| Berea sandstone | 132 | 141 | 6.3 |
| Indiana Limestone | 116 | 141 | 17.7 |
| Tight sandstone | 134 | 141 | 4.9 |
| Shale | 138 | 141 | 2.1 |
| Pentane-heptane-octane mixture – 14.7 psi | | | |
| Berea sandstone | 61 | 66 | 7.5 |
| Indiana Limestone | 74 | 66 | 12.1 |
| Tight sandstone | 83 | 66 | 25.7 |
| Shale | 72 | 66 | 9 |
| Pentane-heptane-octane mixture – 64.7 psi | | | |
| Berea sandstone | 140 | 130 | 7.6 |
| Indiana Limestone | 118 | 130 | 9.2 |
| Tight sandstone | 121 | 130 | 6.9 |
| Shale | 127 | 130 | 2.3 |
